# Why the Cells Look Like That – The Influence of Learning With Emotional Design and Elaborative Interrogations

**DOI:** 10.3389/fpsyg.2018.01653

**Published:** 2018-09-07

**Authors:** Sabrina D. Navratil, Tim Kühl, Steffi Heidig

**Affiliations:** ^1^Psychology of Education, University of Mannheim, Mannheim, Germany; ^2^Hochschule Zittau/Görlitz, University of Applied Sciences, Zittau, Germany

**Keywords:** emotional design, elaborative interrogations, multimedia learning, motivation, cognitive load

## Abstract

We investigated emotional design features that may influence multimedia learning with a self-generated learning (SGL) activity, namely answering elaborative interrogations. We assumed that a positive emotional design would be associated with a higher motivation to accomplish the additional SGL activity. Moreover, an interaction was expected: Learners learning with a positive emotional design should profit from learning with elaborative interrogations whereas learners learning with a negative emotional design would not profit from this strategy to the same extent but would rather benefit through reading. Since no negative emotional design existed yet, we additionally took the challenge to construct one. In a preliminary study, the emotional design features were pre-tested for their influence on emotional state and according to evaluation results, emotional design features were modified for the final versions. For the main study, German students (*N* = 228) were randomly assigned to one of six conditions that resulted from a 3 × 2 Design with emotional design (intended-positive vs. intended-neutral vs. intended-negative) and SGL activity (elaborative interrogations vs. no elaborative interrogations). Contrary to expectations, the intended-negative design worked not out as intended, but was rather comparable with the positive emotional design with respect to learners’ emotional states. Learner motivation was higher when learning with the intended-negative emotional than the neutral design. The quality of the elaborated answers and learner motivation correlated positively with the performance of all learning outcome scores. For transfer questions which addressed the elaborated concepts, an interaction can be reported: learners learning with the positive emotional design benefitted from learning by reading compared to answering the elaborative interrogations. Regarding transfer questions whose concepts were explicitly described in the instructional material, it was better to learn with the intended-negative emotional than the neutral design. According to results of mediation analyses, the influence of motivation on learning outcomes could mostly be explained by the influence of motivation on answering the elaborative interrogations. Implications for creating emotional design as well as its effect on learning are discussed.

## Introduction

How can a multimedia learning environment be designed to positively influence learning? First, one strategy may lie in letting learners generate information on their own. Learning with so-called self-generated learning (SGL) activities means that additional tasks are integrated in the instructional material. Through this, learners may process instructional materials more actively and hence may comprehend the information to be learned more deeply (Dunlosky et al., 2013). Secondly, one essential factor when working with these additional learning tasks may be learners’ emotional states, and associated with this, their motivation. This study addresses the influence of learners’ emotional states induced by an internal induction procedure, namely the usage of emotional design, when learning with a SGL activity, namely the answering of elaborative interrogations, compared to learning without this additional SGL activity.

The main function of SGL activities is the more active processing of instructional material, which consequently may result in better comprehension of the learning issue. According to the *Cognitive Theory of Multimedia Learning* (CTML; Mayer, 2014), it is supposed that there are three essential processes for meaningful learning: (1) actively selecting the relevant information, (2) mentally organizing this information, and (3) integrating the newly acquired information with already existing prior knowledge. Learning with SGL activities may influence especially the second and third of these processes. More precisely, it enhances the organization of selected information from the instructional material to build structural relations among the learned elements by generating correct answers to given elaborative interrogations. Moreover, it forces the learner to integrate the selected material with already existing knowledge in the long-term memory. Within the CTML, conducting these valuable processes is called *generative processing* (Mayer, 2014). In the terminology of the *Cognitive Load Theory* (CLT; Sweller, 1999), this meaningful processing is analogous to the investment of germane resources. The basic assumption of the CLT is the limited capacity of the working memory when dealing with new information. Thereby, two further types of cognitive load have to be distinguished. Whereas intrinsic load is determined by the number of interacting elements and prior knowledge, extraneous load occurs as a result of suboptimal instructional design requiring learners to engage unnecessarily in cognitive processes. A major goal in designing instructional material should on the one hand lie in minimizing extraneous load, while at the same time engaging learners in investigating germane resources (Kirschner, 2002), for instance by using an SGL activity. This means that the more intensive and active work required in processing a SGL activity should be more beneficial than merely reading.

In summary, the fundamental idea of SGL activities is that instead of providing instructional material that merely eases comprehension and processing demands, instructional material may also contain constructive requests which support learning and comprehension. A specific SGL activity, i.e., the answering of elaborative interrogations, will be discussed in more detail in the next paragraph.

Elaborative interrogations (McCrudden and Schraw, 2007), also known as deep reasoning questions (Gholson and Craig, 2006), can be described as integrated ‘why’ and ‘how’ questions within the instructional material (Leinhardt, 2010), that require explanatory responses from learners. When answering them compared to reading only, learners have to infer information that is missing from a text passage. The questions are specifically designed to elaborate the most important information and relations from the learning topic (Roelle et al., 2014). Moreover, monitoring processes are stimulated; when learners realize that they have problems in answering the elaborative interrogations, they may consciously pay more attention to the verbal and pictorial information to understand the learning topic.

Furthermore, the quality of learners’ answers to the elaborative interrogations should be considered (Roelle and Berthold, 2013). One might expect the quality of the answers to be predictive for the performance of the knowledge test questions. According to this expectation, the qualitatively better the elaborative interrogations are answered, the better will be learners’ performances in the knowledge test questions, especially for test questions that address concepts which are asked in the elaborative interrogations and require a deeper understanding of the learning topic (Kühl et al., 2018).

One might expect that additionally answering elaborative interrogations would lead to a higher perception of concentration and mental effort compared to merely reading. Furthermore, this more active and intensive processing could be perceived as more difficult compared to reading a text with pictures. Therefore, elaborative interrogations may only be beneficial as long as working memory resources are not overloaded, so that germane resources can be invested. Hence, it is recommended to keep extraneous load low when learning with the additional task, for instance by providing pictures alongside the text and by constructing well-formulated elaborative interrogations. Moreover, learning with elaborative interrogations may give learners a more realistic estimation of their newly acquired knowledge and a more accurate impression of their performance in a subsequent knowledge test (Mazzoni and Nelson, 1995; Dweck and Elliot, 2005; Kühl et al., 2018).

In sum, elaborative interrogations are ‘knowledge catalysts’ for learning and comprehension because learners are instructed to focus on the most essential information by inferring the important concepts and relations. This should be particularly the case for learning outcomes that are addressed by the elaborative interrogations (Roelle et al., 2015; Kühl et al., 2018).

However, the effectiveness of answering elaborative interrogations might be influenced by individual characteristics of learners, for instance by their emotional state and the associated motivation. In the following paragraph, the term emotional state, its experimental induction and its influence on the learning process will be described in more detail.

Definitions such as mood and emotion seem to be used as synonyms (Heidig et al., 2015) although they should be clearly distinguished. Mood, such as being joyful, is more long-lasting, diffuse, not conscious and mainly independent of a cognitive content (Pekrun, 2006). In comparison, an emotion, such as surprise, is more short-lived, intense and elicited consciously by a cognitive content (Schwarz et al., 1991). A similarity of both constructs is the fact that they can both be described by two dimensions, valence (positive – negative) and activation (activating – deactivating). Because the construct investigated here is rather a combination of characteristics of these two constructs, we would like to refer to it using the term ‘emotional state’ which is already used in a research context (Heidig et al., 2015).

Emotional states can on the one hand be induced externally (e.g., by viewing an emotionally loaded film segment). However, particularly in the context of multimedia learning, where visualizations accompany text, one can also internally induce emotional states by designing the pictures in an emotionally loaded way – a procedure which is termed *emotional design*. The term *emotional design*, first used by Um et al. (2012) in the context of multimedia learning, means the use of various design features that influence learners’ emotional states that in turn have an effect on the comprehension of the learning issue. Thereby, these emotional design features (1) should not comprise additional information to the learning issue and (2) should comprise established characteristics which can influence learners’ emotional states, such as specific color combinations, anthropomorphism, or baby face characteristics.

Regarding color combinations, there are several studies which show that colors are able to influence different states of arousal. Studies could show that warm colors are associated with positive emotional states (e.g., Schaie and Heiss, 1964), with an increase in pleasure and excitement (e.g., Tucker, 1987) and with greater feelings of arousal (e.g., Wolfson and Case, 2000) compared to cool colors. Color combinations such as green–yellow and blue–green lead to higher arousal effects (Cano et al., 2009), whereas combinations such as blue–purple and yellow–red result in a lower arousal effect (Valdez and Mehrabian, 1994). Anthropomorphism is the attribution of uniquely human characteristics, such as additional facial expressions to objects or natural phenomena (DiSalvo and Gemperle, 2003). A study by Dehn and van Mulken (2000) showed that anthropomorphic features are able to increase a person’s attention and engagement with respect to a task compared to when they are not used. Recently, Schneider et al. (2017) reported that anthropomorphism had a positive effect on affective and motivational aspects and led to better performance in a knowledge test. Finally, the baby-face bias is elicited by, for instance, round features and large eyes in objects (Lorenz, 1950). These features seem to be able to induce positive feelings in the learner (Berry and McArthur, 1985).

With respect to the use of emotional design in multimedia learning experiments, the effect on learning processes is seen mostly in the induction of positive emotional states compared to a neutral control condition and consequently focuses on the aesthetically appealing design of multimedia instructional materials. Results of the study by Um et al. (2012) could already show that the use of positive design features compared to a neutral design resulted in improved learning outcomes and higher motivation. To gain a better understanding of the effectiveness of emotional design, Plass et al. (2014) investigated the features of emotional design in more detail. Results revealed that warm colors alone influenced neither learners’ emotional states nor their performance in knowledge tests. But warm colors in combination with round face-like shapes induced a positive emotional state and facilitated comprehension. This result could be explained by enhanced reported motivation; learners learning with the positive emotional design reported higher motivation to learn with the instructional material compared to learners learning with the neutral design.

The *Cognitive-Affective Theory of Learning with Media* (CATLM; Moreno, 2006; Moreno and Mayer, 2007), which is an extension of the previously mentioned CTML, integrates the relation between cognitive and affective processes in multimedia learning. Building on the premises of the CTML, this theory further deals with the influence of motivational and affective aspects during meaningful learning. More precisely, the three processes of selecting, organizing and integrating appear to be mediated by motivational factors that influence learners’ cognitive engagement. Moreover, metacognitive factors might also play a crucial role, which means that learners who are aware of their emotional states and motivation are better able to regulate their learning by planning and monitoring the cognitive processes needed for meaningful learning. Especially when learning with an additional cognitive task, such as answering elaborative interrogations, these processes seem to be even more essential.

However, the CATLM does not provide a complete theory of how emotional states and different learning tasks may interact. Hence, research about emotional states and learning tasks from other fields than multimedia learning will be shortly summarized. A study by Fiedler et al. (1992; Experiment 4) investigated the effect of emotional states on the generation effect. The generation effect can be defined as a memory advantage derived from self-generated information in comparison to read information. When emotional states were induced by an auto-suggestive mood induction procedure, learners were asked to read words or to generate words out of word fragments and to remember them later in a memory task. Results showed that learners who generated the words out of word fragments showed better learning outcomes than learners who merely read the already presented complete words. Moreover, this generation effect was more pronounced in an induced positive emotional state compared to a neutral state. A study by Fiedler et al. (2003; Experiment 3) assessed the effect of a positive or a negative emotional state on the generation effect. After emotional states were induced by viewing emotionally loaded film segments, learners had to learn a list of positive or negative words as either completely formulated or as word fragments which had to be generated. To roughly summarize the results, the authors observed that learners with a positive emotional state showed better recall performances from self-generated information than learners in a negative emotional state, who profited more from the completely formulated words. Similarly, very recently, Schindler et al. (2017) found that learners were better able to remember generated words in a positive compared to a negative emotional state, whereas this was not the case for the recall of completely formulated words. Therefore, it seems that a positive emotional state seems to be more beneficial when an additional learning task requires cognitive flexibility or the generation of new information. If we translate the results of this rather basic research from Fiedler et al. (2003) as well as Schindler et al. (2017) to our study, we can assume that the beneficial effect of elaborative interrogations compared to text is especially pronounced for learners with a positive emotional design, and only to a lesser extent for learners with a neutral design. This can be supported by the explanation that learners in a positive emotional state may especially show increased motivation to engage in a task if confronted with an SGL activity. In contrast, when in a negative emotional state, learners tend to use more rigid and careful types of processing (Bless and Fiedler, 1995) which might improve when learning with text and pictures, rather than with additional elaborative interrogations. As already mentioned regarding the influence of the learner’s emotional state and the generation effect, the study by Fiedler et al. (2003, Experiment 3) showed a reduced recall performance from self-generated information for learners in a negative emotional state. This could be explained by the theory that learners in negative emotional states are more often engaged in emotion-related thoughts or in emotional regulation processes (Gross, 1998) that interrupt the processing of a given cognitive demanding task. It can further be argued that negative emotional states could be detrimental to learner motivation (Pekrun et al., 2007, 2011), and that the motivation factor increases in importance when working with an additional learning task.

All the above-mentioned studies investigated the effect of emotional states on the learning process of text and pictures, but not on learning with a SGL activity, and, where the generation effect was assessed, the emotional state was not induced by emotional design. We accepted this challenge for the current study. We were interested in how different emotional states, induced by emotional designs, may influence learning with answering elaborative interrogations and consequently the learning outcomes. We investigated this by using a 3 × 2 design with emotional design (intended-positive vs. intended-neutral vs. intended-negative) and an SGL activity (elaborative interrogations vs. no elaborative interrogations) as independent variables and retention and a transfer test as dependent variables. In addition to a positive emotional design and a neutral design condition, a negative emotional design condition was added to investigate our research hypotheses. In addition, factors such as learner motivation and cognitive load were taken into account in order to be able to interpret influences and performances regarding the answers to the elaborative interrogations and the learning outcomes. Two preliminary studies were conducted to (1) select the most effective emotional designs for the used pictures and (2) generate well-formulated elaborative interrogations for the SGL activity condition.

In accordance with the theory that different emotional states are associated with varied motivation and this in turn influences the willingness of the learner to engage and to invest additional effort in the learning task, we assumed that learner motivation plays a mediating role. That is, learners with a positive emotional design should report higher motivation to learn the instructional material compared to learners in the other two design conditions (Hypothesis 1a). In addition, if motivation influences the answering of elaborative interrogations, then there should be a systematic connection between learner motivation and the quality of their answers. Consequently, we hypothesized that the more highly the learners are motivated, the better will be the quality of their answers to the elaborative interrogations (Hypothesis 1b).

Furthermore, we expected that the quality of the answers to the elaborative interrogations would be predictive for the learning outcomes. More precisely, the qualitatively higher the answers to the elaborative interrogations, the better the learning outcomes in the knowledge test ought to be, especially for transfer test scores (Hypothesis 2). In sum, Hypotheses 1a, 1b, and 2 were formulated to explain the influence of learners’ emotional states on learning with elaborative interrogations through their motivation and their quality of the answers to the elaborative interrogations. For learners with elaborative interrogations, a combination of Hypotheses 1a, 1b, and 2 can be examined by means of a mediation analysis.

We also hypothesized that learners would invest more effort when learning with elaborative interrogations than when learning with text. Moreover, learners might perceive the additional task as more difficult and more demanding and feel more concentrated compared to when only reading the instructional material. Regarding metacognitive monitoring, we anticipated that learners learning by reading might expect to perform better on the knowledge tests afterwards compared to learners who answered the elaborative interrogations (Hypothesis 3).

Finally, we hypothesized an interaction between emotional design and SGL-activity: learning with elaborative interrogations compared to learning through reading should be especially beneficial with a positive emotional design, and only to a lesser extent with a neutral design. In contrast, learners learning with a negative emotional design would not be expected to profit from learning with elaborative interrogations as much as from learning without this additional SGL activity (Hypothesis 4).

## Materials and Methods

### Preliminary Study: Emotional Design

**FIGURE 1:**
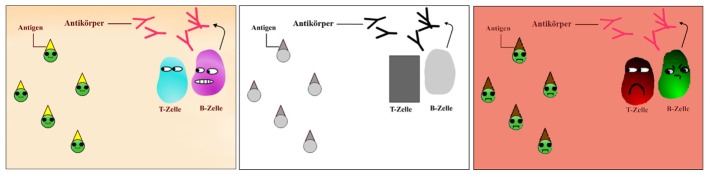
Pictures of emotional design variations of the multimedia learning environment: Intended-positive emotional design **(left)**, intended-neutral design **(middle)**, intended-negative emotional design **(right)**.

A multimedia learning environment developed by Plass et al. (2014) consisting of an animation accompanied by narration, served as a basis for the instructional material. Ten pictures (key frames) taken from this material were used in the preliminary study in order to identify the appropriate designs (color combinations, anthropomorphisms, baby-face features) of the instructional material, particularly for the newly designed intended-negative emotional design condition, but also for the intended-positive emotional design condition. For each of the ten pictures taken from the original instructional material, one to three variations of the intended-negative and the intended-positive emotional design were presented, consisting of different colors and shapes, along with the appropriate picture for the intended-neutral condition each time as a control. Whereas in the case of the intended-negative emotional design, background colors (different bright and saturated red hues) and different negative emotional expressions (angry eyes and shapes of eyebrows) were varied, in the intended-positive emotional design, mainly friendly facial expression features (e.g., different smiles) were varied. The pictures for the intended-neutral condition were the pictures from the original learning environment. Examples of pictures chosen for the three versions used are shown in **Figure 1**: (1) Intended-positive: warm and bright hues, round face-like shapes and happy facial expressions, (2) intended-neutral: gray-scale and square shapes and (3) intended-negative: darker hues, red background, round face-like shapes and angry facial expressions. 57 learners were instructed to look carefully at all picture variations and rank them from the most positive to the most negative one (the higher the value, the more negative the rank). For all ten pictures, the most negative, neutral and positive ranked versions were summed up in three separate scores. A non-parametric Friedman test for repeated measures was conducted and rendered a Chi-square value of 90.41 which was significant (*p* < 0.001). That is, the ten most positive pictures were significantly ranked as more positive (*Mdn* = 13.00) compared to the ten neutral (*Mdn* = 28.00) or the ten negative pictures (*Mdn* = 44.00). This relationship was also true when comparing separately for each picture the rankings of the most intended-positive with the intended-neutral and the most intended-negative chosen picture. For nine of the ten chosen pictures, the ranking of the three versions differed significantly from each other (all *p*_s_ < 0.05), with the exception of one picture, where the comparison of an intended-negative with an intended-neutral design failed to reach statistical significance (but descriptively was still in the consistent direction). In total, 22 pictures were used in the whole instructional material. Based on these results of the ten investigated pictures, the remaining images were adapted according to their design features.

### Participants and Design

A total of 260 students with various educational backgrounds from a German university volunteered to participate in return for either course credit or payment in this study. The data of 32 students had to be omitted from further analyses (23 students participated twice due to an error in the recruitment, six students showed mainly missing values due to computer problems, one did not fill out the knowledge test, one studied biology and showed high prior knowledge and one had spoken German for less than 3 years and had severe problems understanding the instructions in the instructional material and the knowledge test questions). Of the remaining 228 learners, 184 were female and 44 were male (average age: 22.1 years). They were randomly assigned to one of six conditions, which resulted from a 3 × 2 design with emotional design (intended-positive vs. intended-neutral vs. intended-negative) and SGL activity (elaborative interrogations vs. no elaborative interrogations) as independent variables. 34–40 learners were assigned to each condition (see **Table 1**). A single session lasted about 45 min.

**Table 1:** Means (*SD*) of the control variables, the manipulation check, the quality of the answers of the elaborative interrogations in the respective SGL activity conditions, the cognitive load scores, the reported motivation and the performances in the retention and transfer test in the six experimental conditions.

Emotional state	Intended-positive		Intended-neutral		Intended-negative	
SGL activity	No elaborative interrogations (*n* = 38)	Elaborative interrogations (*n* = 38)	No elaborative interrogations (*n* = 34)	Elaborative interrogations (*n* = 38)	No elaborative interrogations (*n* = 40)	Elaborative interrogation (*n* = 40)
**Control variables**						
Self-reported prior knowledge	1.87 (1.71)	1.82 (1.43)	1.32 (1.45)	1.82 (1.67)	1.78 (1.35)	1.53 (1.11)
Prior knowledge (open question)	0.55 (1.13)	0.74 (1.45)	0.56 (1.42)	0.45 (0.92)	0.43 (0.90)	0.38 (0.84)
Spatial abilities	5.05 (3.42)	4.76 (3.73)	3.59 (3.64)	3.63 (3.51)	4.03 (4.18)	4.38 (3.35)
**Emotional state check: before learning**						
Emotional state	5.53 (1.62)	5.39 (1.85)	5.26 (1.76)	5.13 (1.70)	5.75 (2.00)	5.58 (1.53)
**PANAVA-KS**						
Valence	6.26 (1.73)	6.21 (1.90)	5.84 (1.65)	5.49 (1.78)	6.56 (1.85)	6.11 (1.41)
Positive activation	5.12 (1.39)	4.89 (1.45)	4.63 (1.22)	4.70 (1.23)	5.18 (1.44)	4.95 (1.31)
Negative activation	3.81 (1.38)	4.13 (1.38)	4.16 (1.41)	4.27 (1.44)	3.74 (1.45)	3.88 (1.33)
**Emotional state check: after learning**						
Emotional state	5.66 (1.58)	5.39 (1.53)	4.91 (1.66)	4.63 (1.70)	5.68 (1.86)	5.23 (1.53)
**PANAVA-KS**						
Valence	5.86 (1.50)	5.91 (1.59)	5.21 (1.57)	4.88 (1.55)	6.19 (1.62)	5.59 (1.57)
Positive activation	5.08 (1.48)	4.99 (1.33)	4.34 (1.39)	4.41 (1.31)	5.08 (1.44)	4.98 (1.33)
Negative activation	3.86 (1.34)	4.33 (1.34)	4.26 (1.35)	4.79 (1.56)	3.77 (1.42)	4.31 (1.39)
**Performance of elaborative interrogations**						
Mentioned core elements	–	3.97 (0.85)	–	3.61 (1.26)	–	3.95 (1.01)
**Cognitive load**						
Effort	4.66 (1.12)	4.68 (1.49)	4.24 (1.54)	4.53 (1.48)	5.08 (1.54)	4.68 (1.54)
Difficulty	3.00 (1.51)	3.32 (1.63)	3.76 (1.50)	3.74 (1.59)	2.98 (3.63)	3.63 (1.71)
Concentration	4.68 (1.19)	4.87 (1.17)	4.44 (1.73)	4.50 (1.23)	4.90 (1.57)	4.78 (1.59)
Estimated success	4.05 (1.14)	3.89 (1.56)	3.44 (1.31)	3.53 (1.27)	4.10 (1.28)	3.43 (1.47)
**Motivation**						
Motivation	34.61 (12.48)	34.55 (11.19)	32.76 (10.98)	31.21 (11.15)	38.35 (9.84)	35.33 (13.65)
**Learning outcomes**						
*Retention*						
Interrogation	7.50 (2.17)	7.34 (1.89)	7.24 (1.97)	7.24 (2.27)	7.33 (2.16)	7.63 (2.00)
NoInterrogation	6.63 (1.84)	6.08 (1.58)	6.18 (1.57)	5.68 (2.36)	6.13 (1.91)	6.15 (1.63)
*Transfer*						
Interrogation	5.24 (1.34)	4.21 (1.34)	4.88 (1.34)	4.92 (1.48)	4.73 (1.77)	4.93 (1.77)
NoInterrogation	3.97 (1.82)	3.74 (1.33)	3.47 (1.40)	3.29 (1.59)	3.93 (1.75)	4.03 (1.83)

### Design of Instructional Material

A multimedia learning environment on the topic of immunization was presented on nine pages and dealt specifically with immunization phases and active and passive immunization. It was learner-controlled and learners could move backward and forward within the instructional material on the computer.

Emotional design was presented in three different conditions. Whereas the intended-positive emotional design was characterized by warm color hues, round shapes, laughing mouths, and big eyes, the intended-neutral design was black and white and consisted of simple geometric shapes, and the intended-negative emotional design had a red background with dark and clashing color hues, round shapes and angry/sad expressions (cf. **Figure 1**).

**FIGURE 2:**
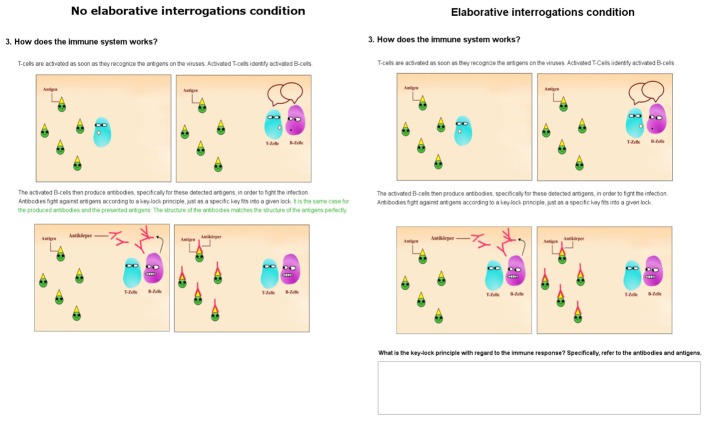
Examples of an instructional material page in the intended-positive emotional design for both SGL activity conditions. **(Left)** No elaborative interrogations condition (letters highlighted in green are for illustrational purposes only). **(Right)** Elaborative interrogations condition. The condition titles are also for illustration purposes only, but were not presented in the instructional material. The original instructional material was in German.

Considering the factor SGL activity, learners were assigned either to the condition with elaborative interrogations or to a condition with text.^1^ The two conditions differed in the number of words because information which had to be generated by the elaborative interrogations was not provided in the text itself, whereas in the text condition all the information was provided (highlighted green in **Figure 2**). Whereas the elaborative interrogations condition consisted of 541 words, the condition without elaborative interrogations consisted of 633 words. The title of each paragraph was always at the beginning of each page. There then followed a text passage and one to four corresponding pictures. For learners in the group without the elaborative interrogations condition, there was no direct instruction, except to learn the instructional material. In the elaborative interrogations condition group, learners were additionally instructed to answer the elaborative interrogations given at the bottom of five of the nine pages. The elaborative interrogations were mandatory questions in order to reach the next learning page. Examples of a learning page of the intended-positive emotional design in groups with the independent variable SGL activity can be seen in **Figure 2**.

### Measures

The measures consisted of (1) a spatial ability test, (2) prior knowledge questions, (3) two emotional state manipulation checks, (4) items assessing cognitive load, (5) a motivation questionnaire, (6) a knowledge test (retention and transfer), (7) a demographical questionnaire and (8) an evaluation sheet about the study and its contents.

#### Spatial Ability Test

A shortened version of the Paper Folding Test (PFT; Ekstrom et al., 1976) with 10 items was used to investigate learners’ spatial abilities. They had to complete it in 3 min. One point was given for each correct answer and one point was subtracted for each incorrect answer. This leads to a theoretical minimum of -10 points and a maximum of 10 points.

#### Prior Knowledge Test

Adapted from Plass et al. (2014), learners were asked to rate their knowledge of immunization on a 5-point scale (from ‘very low to ‘very high’). Learners had to assess their knowledge about relevant immunization topics in a 7-item self-report checklist (Park et al., 2015), for instance ‘I can explain what antibodies are.’ The answer on the 5-point scale and the number of *yes* answers on the self-report checklist were summed up as a score for ‘self-reported prior knowledge.’ In addition, an open question was presented (‘In case you already possess prior knowledge concerning the immunization: please write down everything you know concerning the issue ‘immunization”). A coding scheme for scoring learners’ answers was developed. Overall, learners’ prior knowledge was low (*M* = 1.69, *SD* = 1.46) and only 54 of 228 learners (23.7%) tried to answer the open question.

#### Emotional State Check

One item defined as ‘emotional state’ with the adjectives ‘depressed’ to ‘joyful’ had to be estimated on a 9-point scale. Additionally, the 10 items of the PANAVA-KS (Schallberger, 2005) were used to check learners’ emotional states. All items had to be answered on a nine-point scale directly before (as a baseline check) and after the learning phase. The verbal anchors were only presented for the extremes and the neutral position (e.g., ‘very unhappy’ – ‘neutral’ – ‘very happy’). The questionnaire included two items for valence and four items each for the positive and the negative activation level, which were summed up to three separate total scores (valence; positive activation; negative activation).

#### Cognitive Load Items

Four items were used to assess learner’s cognitive load. Learners were asked to rate their subjective perceived invested mental effort, concentration, difficulty of instructional material, and estimated success in a subsequent knowledge test on a seven-point scale (from ‘not at all’ to ‘very high’).

#### Motivation

For the self-reported measure of motivation, a questionnaire developed by Isen and Reeve (2005) was used. Learners were asked to rate their motivation to learn with the instructional material on eight items on a seven-point scale (from ‘strongly disagree’ to ‘strongly agree’).

#### Knowledge Test

Learning outcomes were measured by means of (1) 18 retention multiple choice questions and (2) five open transfer questions. The computer-based multiple choice questions measured learners’ understanding of the most essential concepts of the instructional material. For example, it was asked, ‘Which cells are producing antibodies? (a) Macrophages, (b) Phagocytes, (c) B-cells, (d) T-cells, or (e) Antibodies’. There was always one correct answer for each multiple choice question and learners could always only select one answer. Learners received one point for each correct answer, and no negative points were given. All five open transfer questions were paper-based and each question was presented on one page with an appropriate time limit which was presented next to the knowledge test question. Moreover, learners were informed via headphones about each time limit and when they had to turn to the next page. The transfer test assessed learners’ ability to apply the learned concepts to solve problems. For example, it was asked, ‘HIV (Human Immunodeficiency Virus) destroys T-cells in immune system. Explain the consequences of this infection by describing the role of T-cells in the process of the immune system.’ Each transfer question was scored according to a coding scheme that consisted of a list of possible correct answers. Learners received one point for each correct aspect named for each question. The final score for transfer was determined by adding up the points from all transfer questions. The questions from the retention and transfer test were divided into questions addressing concepts which had to be generated by elaborative interrogations (‘Retention_Interrogation, Transfer_Interrogation’), and questions addressing concepts which were already provided in the text (‘Retention_NoInterrogation, Transfer_NoInterrogation’).

All transfer questions were scored by two independent raters who had no prior knowledge of the experimental conditions. Inter-rater reliability was estimated utilizing a correlational analysis. The inter-rater reliability estimation for the transfer test indicated moderate agreement (*r* = 0.77, *p* < 0.001). Cases of disagreement were resolved by reaching a consensus.

### Procedure

An ethics approval was not required for this research as per institutional and national guidelines. By the time we conducted the study and acquired the data, it was neither compulsory nor customary at the respective university to seek explicit ethical approval for an experimental study including only participants’ self-reports on their emotional states. However, we carefully ensured that the study was conducted in line with the ethical guidelines of the American Psychological Association and in full accordance with the ethical guidelines of the German Psychological Society. The study exclusively made use of anonymous questionnaires and the data was coded by using codenames only. Written informed consent was obtained according to the guidelines of the German Psychological Society and included information about (1) research object, (2) study procedure, (3) duration and allowance, (4) possible benefits of participation, (5) anonymity of data collection, and (6) possible risks of participation. Participants were, furthermore, explicitly informed that participation was voluntary and could be terminated at any time without any reason or negative consequences for the participant. Finally, participants had to declare that they had read the informed consent information, and agreed to the rules of participation.

In each test session, up to six learners for the same SGL activity condition were seated behind a table with a privacy shield partitioned into six ‘viewing booths’ with individual computers. Hence, learners could not see other learners during the study. At first, all learners were given the spatial ability test. After being provided with a brief overview of the study procedure, learners had to fill out the prior knowledge questionnaire and were asked to fill out the first manipulation check. Immediately after this, one of the six conditions of the multimedia instruction was presented on the computer. After completing the learning phase, learners were asked to fill out the manipulation check again, along with the cognitive load items and the motivation questionnaire. Then, the retention multiple choice questions were presented and learners were instructed to answer them. Finally, learners were asked to take the paper-based open transfer questions out of a folder on their desk and answer them. After the completion of the transfer test, it was placed back into the folder and a further instruction on the computer asked learners to fill out the evaluation sheet. The final screen informed learners that they had just completed the experiment and instructed them to fill out demographical data to receive their reimbursement.

## Results

### Control Variables

Separated two-factor ANOVAs with emotional design (intended-positive vs. intended-neutral vs. intended-negative) and SGL activity (elaborative interrogations vs. no elaborative interrogations) revealed no differences for emotional state or SGL activity, and no interaction for the two control variables of learners’ self-reported prior knowledge and the performance of the open question on prior knowledge (all *F*_s_ < 1, all *p*_s_ > 0.10). For the third control variable, spatial ability, an ANOVA showed a marginally significant main effect of emotional design [*F*(2,222) = 2.35, *p* = 0.097, $\eta_{p}^{2}$ = 0.02]. Bonferroni-corrected pairwise comparisons revealed that learners learning with the intended-positive emotional design showed higher spatial abilities than learners learning with the intended-neutral design (*p* = 0.099), while the intended-negative emotional design did not differ from the other two designs (both *p*_s_ > 0.10). When using spatial ability as a covariate, the same pattern of results occurred. Hence, for the further analyses, we refrained from using this covariate. All descriptive data is presented in **Table 1**.

### Emotional Design Induction

Learners had to rate their emotional states before learning the instructional material, as a baseline. Regarding the question about their emotional states, a two-factor ANOVA showed no significant effects (all *F*_s_ < 1, all *p*_s_ > 0.10). With respect to the PANAVA-KS items, two-factor ANOVAs showed no significant main effects and no interaction for the positive as well as the negative activation scores (all *F*_s_ < 1, all *p*_s_ > 0.10). Regarding the valence, there was no significant main effect for SGL activity and no interaction (all *F*_s_ < 1, all *p*_s_ > 0.10). There was, however, a significant main effect of emotional design [*F*(2,222) = 3.23, *p* = 0.04, $\eta_{p}^{2}$ = 0.03]: Bonferroni-corrected pairwise comparisons revealed that learners who received the intended-neutral design afterwards reported lower valence scores (*M* = 5.65, *SD* = 1.71) compared to learners who received the intended-negative emotional design (*M* = 6.34, *SD* = 1.65), *p* = 0.05. The intended-positive emotional design (*M* = 6.24, *SD* = 1.80) did not differ from the other two designs, both *p*_s_ > 0.10.

For all emotional state scores after the learning phase, the corresponding scores before the learning phase are used as covariates, since there were pre-experimental differences. After the learning phase, regarding the one question about emotional states, a main effect of emotional design can be reported [*F*(2, 221) = 6.30, *p* = 0.002, $\eta_{p}^{2}$ = 0.05]. Bonferroni-corrected pairwise comparisons revealed that the intended-positive emotional design led to higher positive values (*M* = 5.53; *SD* = 1.55) than the intended-neutral design (*M* = 4.76, *SD* = 1.67), *p* < 0.001. The intended-negative emotional design (*M* = 5.45, *SD* = 1.71) did not differ from the other two designs, both *p*_s_ > 0.10. A marginally significant main effect of SGL activity could be reported, too, [*F*(1,221) = 3.01, *p* = 0.08, $\eta_{p}^{2}$ = 0.01]: Learning with text (*M* = 5.44, *SD* = 1.73) showed higher positive values than learning with elaborative interrogations (*M* = 5.09, *SD* = 1.61), *p* = 0.08. No significant interaction was observed. With respect to the valence scores of the PANAVA-KS, a main effect of emotional design was revealed [*F*(2,221) = 3.75, *p* = 0.03, $\eta_{p}^{2}$ = 0.03]. Bonferroni-corrected pairwise comparisons revealed that the intended-positive (*M* = 5.88, *SD* = 1.54), *p* = 0.04, and the intended-negative emotional design (*M* = 5.89, *SD* = 1.61), *p* = 0.08, differed significantly in higher positive valence compared to the intended-neutral design (*M* = 5.03, *SD* = 1.56). No significant main effect of SGL activity or interaction could be reported. Regarding positive activation, a significant main effect of emotional design was demonstrated [*F*(2,221) = 4.01, *p* = 0.02, $\eta_{p}^{2}$ = 0.04]. Bonferroni-corrected pairwise comparisons revealed that the intended-positive (*M* = 5.03, *SD* = 1.40), *p* = 0.03, and the intended-negative emotional design (*M* = 5.03, *SD* = 1.38), *p* = 0.06, differed significantly in higher positive activation compared to the intended-neutral design (*M* = 4.38, *SD* = 1.34). No significant main effect of SGL activity nor interaction could be reported. Regarding negative activation, a significant main effect of SGL activity was observed [*F*(1,221) = 8.65, *p* = 0.004, $\eta_{p}^{2}$ = 0.04]: learning with elaborative interrogations (*M* = 4.47, *SD* = 1.44) showed higher negative activation compared to learning by reading (*M* = 3.95, *SD* = 1.38), *p* = 0.004. No significant main effect of emotional design nor an interaction could be shown (all *F*_s_ < 1, all *p*_s_ > 0.10). Since the negative emotional design did not work in terms of the described characteristics, but was in some ways comparable with the positive emotional design, it is referred to as ‘intended-negative’ emotional design.

### Motivation

According to Hypothesis 1a, we anticipated that learners’ different emotional states would be associated with varied motivation. A 3 × 2 ANOVA with the independent variables SGL activity and emotional design were conducted and yielded a significant main effect of emotional design [*F*(2,222) = 3.29, *p* = 0.04, $\eta_{p}^{2}$ = 0.03]. Bonferroni-corrected pairwise comparisons revealed that an intended-negative emotional design leads to higher motivation (*M* = 36.84, *SD* = 11.92) than the neutral design (*M* = 31.94, *SD* = 11.02), *p* = 0.03, whereas the positive design (*M* = 34.58, *SD* = 11.77) did not significantly differ from the intended-negative and neutral design, both *p*_s_ > 0.10. Neither a significant main effect of SGL activity, nor an interaction was observable (all *F*_s_ < 1, all *p*_s_ > 0.10).

Furthermore, a correlational analysis was performed to verify Hypothesis 1b that assumed a relation between motivation and the quality of learners’ answers to the elaborative interrogations. For the correlational analyses, only the 116 learners in the elaborative interrogations condition were considered. Results showed that learner motivation correlates significantly with the quality of the answers to the elaborative interrogations, *r* = 0.31, *p* = 0.001.

Regarding learning outcomes, results revealed significant relations between learner motivation and their performance in all knowledge tests: Retention_Interrogation (*r* = 0.24, *p* < 0.001), Retention_NoInterrogation (*r* = 0.21, *p* = 0.001), Transfer_Interrogation (*r* = 0.18, *p* = 0.005) and Transfer_NoInterrogation (*r* = 0.22, *p* = 0.001).

### Quality of Answers of Elaborative Interrogations

The basic idea of Hypotheses 1a, 1b, and 2 is that different emotional designs influence the quality of the answers differently. However, results revealed no influence of emotional designs on the quality of the answers to elaborative interrogations (all *F*_s_ < 1, all *p*_s_ > 0.10).

Furthermore, it was assumed that the quality of the answers is predictive for learners’ performances in the knowledge test questions (Hypothesis 2). Again, only the 116 learners in the elaborative interrogations condition were considered for the four correlational analyses. The performance of answering elaborative interrogations correlated significantly with all knowledge test scores: Retention_Interrogation, *r* = 0.33, *p* < 0.001, Retention_NoInterrogation, *r* = 0.26, *p* = 0.006, Transfer_Interrogation, *r* = 0.27, *p* = 0.003, and marginally with Transfer_NoInterrogation, *r* = 0.17, *p* = 0.07.

Because results revealed an influence of learner motivation on the quality of the answers of the elaborative interrogations as well as all knowledge test questions, and the quality of the answers of the elaborative interrogations are predictive for all knowledge test questions, four separate mediation analyses with respect to the four learning outcome scores were conducted. For doing this, the SPSS-macro Process v2.15 was used (Hayes, 2013) with 5.000 bootstrap samples. Regarding Retention_Interrogation, there was a significant indirect effect of learner motivation on the learning outcome through the quality of learners’ answers of the elaborative interrogations, *b* = 0.01, BCa CI [0.004, 0.028]. However, this can only be defined as a partial mediation because of a significant direct effect, too, *b* = 0.04, BCa CI [0.008, 0.069]. Regarding Retention_NoInterrogation, there was a significant indirect effect of learner motivation on the learning outcome through the quality of learners’ answers of the elaborative interrogations, *b* = 0.01, BCa CI [0.002, 0.025], with no significant direct effect, *b* = 0.02, BCa CI [-0.010, 0.048] remaining. With respect to Transfer_Interrogation, there was a significant indirect effect of learner motivation on the learning outcome through the quality of learners’ answers of the elaborative interrogations, *b* = 0.01, BCa CI [0.002, 0.022], with no significant direct effect, *b* = 0.02, BCa CI [-0.009, 0.040] remaining. And regarding Transfer_NoInterrogation no significant indirect effect could be revealed.

### Cognitive Load

According to Hypothesis 3, we anticipated differences in cognitive load between learning with elaborative interrogations and learning through reading. A 3 × 2 ANOVA with the independent variables SGL activity and emotional design and the dependent variables effort, concentration, perceived difficulty of the instructional material, and estimated success in a subsequent knowledge test were conducted.

Regarding effort and concentration, there were neither main effects for emotional design, nor for SGL activity and no interaction of both factors was observed (all *F*_s_ < 1, all *p*_s_ > 0.10). With respect to difficulty, a marginally significant main effect of emotional design could be shown [*F*(2,222) = 2.82, *p* = 0.06, $\eta_{p}^{2}$ = 0.03]. Bonferroni-corrected pairwise comparisons revealed that the neutral design (*M* = 3.75, *SD* = 1.54) led to higher perceived difficulty than the positive emotional design (*M* = 3.16, *SD* = 1.57), *p* = 0.07. The intended-negative emotional design did not differ from the other two designs (*M* = 3.30, *SD* = 1.64), both *p*_s_ > 0.10. Neither a significant main effect of SGL activity nor an interaction could be found (all *F*_s_ < 1, all *p*_s_ > 0.10). With respect to learner’s estimated success in the subsequent knowledge test questions, results showed a marginally significant main effect of emotional design, *F*(2,222) = 2.47, *p* = 0.09, $\eta_{p}^{2}$ = 0.02. Bonferroni-corrected pairwise comparisons revealed that the positive emotional design (*M* = 3.97, *SD* = 1.36) led to the perception of a better performance in a subsequent knowledge test compared to learning with the neutral design (*M* = 3.49, *SD* = 1.28), *p* = 0.08, while the intended-negative emotional design (*M* = 3.76, *SD* = 1.41) did not differ from the positive or neutral design (both *p*_s_ > 0.10). Neither a significant main effect of the SGL activity nor an interaction could be demonstrated (both *F*_s_ < 1, both *p*_s_ > 0.10).

### Learning Outcomes

Hypothesis 4 dealt with the main focus of this study, the interaction of learners’ emotional states and learning with an SGL activity. We anticipated that emotional design would interact with the SGL activity in such a way that learners with the positive emotional design in particular would profit from the answering of elaborative interrogations compared to the other two emotional design conditions, while learners with the negative emotional design would profit more from the use of text and pictures compared to answering elaborative interrogations.

Concerning retention, for the Retention_Interrogation (which is comprised of all concepts that were addressed by the elaborative interrogations) as well as for the Retention_NoInterrogation score (which is comprised of all concepts which were presented in the text), the 3 × 2 ANOVAs yielded neither a significant main effect of SGL activity nor of emotional design nor a significant interaction between the factors (all *F*_s_ < 1, all *p*_s_ > 0.10).

Regarding Transfer_Interrogation (this total score is comprised of questions that asked for concepts which were addressed by the elaborative interrogations), a 3 × 2 ANOVA yielded a significant interaction of both factors [*F*(2,222) = 3.64, *p* = 0.03, $\eta_{p}^{2}$ = 0.03]. Other than anticipated, learners learning with the positive emotional design performed better when learning by reading (*M* = 5.23, *SD* = 1.34) compared to answering elaborative interrogations (*M* = 4.21, *SD* = 1.34), *p* = 0.004. Learners with a neutral design and learners with the intended-negative emotional design performed equally well with elaborative interrogations and with text (both *p*_s_ > 0.10). Neither a main effect of emotional design nor of SGL activity was observable (both *F*_s_ < 1, both *p*_s_ > 0.10). With respect to Transfer_NoInterrogation (this total score is comprised of questions that asked for concepts which were presented in the instructional material), a 2 × 3 ANOVA yielded a marginally significant main effect of emotional design, *F*(2,222) = 2.74, *p* = 0.07, $\eta_{p}^{2}$ = 0.02. Bonferroni-corrected pairwise comparisons revealed that learners learning with the intended-negative emotional design (*M* = 3.98, *SD* = 1.78) showed better performances than learners learning with a neutral design (*M* = 3.38, *SD* = 1.50), *p* = 0.08, while learning with the positive emotional design (*M* = 3.86, *SD* = 1.59) did not differ from the other two designs (both *p*_s_ > 0.10). Neither a main effect of emotional design nor an interaction between both factors was observable (all *F*_s_ < 1, all *p*_s_ > 0.10).

**FIGURE 3:**
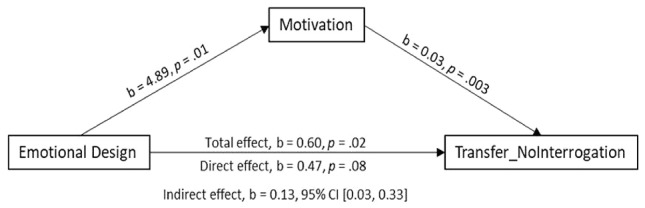
Differences in Transfer_NoInterrogation questions for emotional design (Contrast 2: intended-negative vs. neutral). mediated by learner motivation.

As outlined, learner motivation might have a mediating role between learners’ emotional states and learning outcome. Results revealed that learners learning with an intended-negative emotional design reported significantly higher motivation than learners learning with the neutral design. Furthermore, learners learning with the intended-negative emotional design showed better performances in the Transfer_NoInterrogation test than learners learning with the neutral design. From this, it would be possible to argue that learner motivation might be a mediator between learners’ emotional states and the influence on the learning outcome in Transfer_NoInterrogation. To further investigate this claim, a mediation analysis was performed, with Transfer_NoInterrogation as dependent variable, emotional state as independent variable and learner motivation as mediator. For this, the SPSS-macro Process v2.15 was used (Hayes, 2013) with 5,000 bootstrap samples. Given that emotional states as predictor was a multicategorical variable with three levels, two dummy-coded variables were created to conduct the mediation analysis (Hayes and Preacher, 2014) with the neutral design condition as the reference group, and the positive emotional design (coded 1) defined as Contrast 1, and the intended-negative emotional design (coded -1) as defined as Contrast 2. Consistent with the results of the 3 × 2 ANOVA reported above, the effect of Contrast 1 on motivation was not significant (*p* > 0.10), whereas the effect of Contrast 2 was significant, *b* = 4.89, *t*(225) = 2.60, *p* = 0.01. Similarly, the effect of Contrast 1 on the performance of the Transfer_NoInterrogation was marginally significant [*b* = 0.48, *t*(225) = 1.79, *p* = 0.08], and the effect of Contrast 2 was significant, *b* = 0.60, *t*(225) = 2.27, *p* = 0.02. Moreover, when learner motivation and the two contrasts were entered simultaneously into the model predicting the performance of the Transfer_NoInterrogation, the effect of motivation was significant, *b* = 0.03, *t*(224) = 2.97, *p* = 0.003. Importantly, whereas the direct effect of Contrast 1 (i.e., neutral vs. positive) remained non-significant (*p* > 0.10), the direct effect of Contrast 2 (i.e., neutral vs. intended-negative) was reduced, *b* = 0.47, *t*(224) = 1.76, *p* = 0.08. With respect to the indirect effect, for Contrast 1 it was not significant, but for Contrast 2 a significant indirect effect of emotional design on the performance of the Transfer_NoInterrogation through learner motivation can be shown, *b* = 0.13, 95% CI [0.03, 0.33]. **Figure 3** summarizes the results for Contrast 2.

## Summary and Discussion

### Summary of Results

The present study investigated the influence of emotional states, induced by emotional design, on learning using the strategy of answering elaborative interrogations. Emotional states were thought to be related to motivation which might support the accomplishment of the additional SGL activity. Results revealed that learners learning with the intended-negative emotional design had higher motivation than learners learning with the neutral design, and the positive emotional design had no effect. A systematic relation between learner motivation and the quality of the answers of the elaborative interrogations and learning outcomes could be observed. The higher the motivation of the learners, the qualitatively higher their answers to elaborative interrogations and the better they were in the knowledge tests. Moreover, the quality of the answers to the elaborative interrogations was predictive for the knowledge test performance: The qualitatively higher the answers, the better the performance of the knowledge test questions. Finally, as the mediation analyses revealed, the influence of motivation on learning outcomes could mostly be explained by the influence of motivation on answering the elaborative interrogations.

With respect to cognitive load, while the neutral design was perceived as more difficult as the intended-negative emotional design, learning with the positive emotional design led to increased estimation of likely success in a subsequent knowledge test compared to the neutral design, even though this was not the case. Finally, whereas for both retention scores no effects could be found, an interaction regarding transfer questions whose concepts had to be generated by the elaborative interrogations could be revealed. Other than expected, the positive emotional design supported learning by reading compared to answering elaborative interrogations. With respect to transfer questions whose concepts could be derived from the text, a main effect of emotional design can be reported: the intended-negative emotional design led to better performances than the neutral design, while the positive emotional design had no effects. This effect of the negative emotional design could be explained by the corresponding motivation this design elicited in learners.

### Emotional Design

The internal induction by using emotional designs worked only in part. The one emotional state question as well as the valence score of the PANAVA-KS showed that the positive emotional design was more positive than the neutral design. However, contrary to our expectations, the intended-negative design was not perceived as more negative (as in the preliminary study) compared to the other two designs.

Surprisingly, with respect to the positive activation of the PANAVA-KS, the positive and the intended-negative emotional design did not differ but were considered as more positively activating than the neutral design. This is partly in line with several studies using a similar instructional material (only comparison of the positive emotional and the neutral design: Um et al., 2012; Plass et al., 2014; Park et al., 2015). With respect to the intended-negative emotional design, this was characterized by different colors and anthropomorphisms, which in the hindsight might be perceived as more vivid and interesting and thus might direct learners’ attention more to the instructional material (Dehn and van Mulken, 2000). In comparison, the black and white hues of the neutral design without any colors might be perceived as less interesting and less motivating to learn with, which leads to less attention. This theory can be supported by the outcome that learners learning with the intended-negative emotional design reported higher motivation to learn with the instructional material than those with the neutral design.

An important point to mention here is that there may have been a difference in learners’ perception of pictures between a within-subject design (as in the preliminary study) and a between-subject design (as in the main study). In the preliminary study, they had the opportunity to compare all three different designs, while in the main study only one emotional design condition was presented to each learner. Perhaps the intended-negative emotional design was perceived as very negative in the preliminary study in comparison to the other two conditions, but not in the main study, as no direct comparison with the other emotional design conditions was possible. Furthermore, from a methodological perspective, it should be mentioned that in the preliminary study of emotional designs, only the pictures were ranked and no additional learning text was provided. Thus, the results of this preliminary study only allows us to report on how the pictures *per se* were perceived by the participants, but not in combination with a learning text as was the case in the main study. This also corresponds with the argument that the intended-negative emotional design, in respect to its emotional features, should be coherent with the content of the learning text. If the instructional material discusses an unpleasant topic, such as immunization, and in addition it might be perceived as not pleasantly but rather, unpleasantly designed, then learners may feel coherence. Thus, learners may feel it is enjoyable to learn with this material because everything fits together. Again, the result that learners learning with the intended-negative emotional design reported higher motivation to learn with the instructional material than learners learning with the neutral design supports this theory. Nevertheless, this interpretation is notional and should be addressed in future studies.

Furthermore, results showed that learning with elaborative interrogations led to higher negative activation compared to only reading. This could be explained with the theory that learning with the additional cognitive demanding task may be perceived as more demanding and hence somewhat annoying compared to learning with an instruction only to read and understand the instructional material. The result of the emotional state question supports this explanation in showing that learning with text was perceived as more positive than learning with elaborative interrogations.

In conclusion, it could be shown that the positive emotional design led to a more positive emotional state than the neutral design. However, regarding the intended-negative emotional design, it is obviously more complicated to create a negative emotional state by mirroring and adapting design features from the positive emotional design. Since we had already manipulated the colors and the anthropomorphisms to induce a negative emotional state, the question arises as to which additional factors would have had to be changed to induce a negative emotional state successfully. One approach for a further study could be the investigation of coherence. Could it be that the coherence between the emotional design features and the learning content plays a crucial role in how positively or negatively the instructional material is perceived and is able to induce the desired emotional states? Two instructional materials with coherent emotional design features vis-à-vis their content could be tested against each other.

### Motivation

It was assumed that learning with the positive emotional design would lead to higher motivation compared to the other two conditions. However, results showed that the positive emotional design had no influence on learner motivation, but instead the intended-negative emotional design led to higher motivation than the neutral design. Since the neutral design consisted of only black and white hues and geometrical forms, one might assume that motivation cannot be elicited from it. But, in case of learning with the intended-negative emotional design, it might be that the congruity between the emotional design and the learning topic elicits higher motivation to learn the instructional material. More specifically, a coherent emotional design, such as bad-looking eyes of the cells, clashing color combinations and a red background, makes the instructional material more interesting and motivating in terms of learning. In comparison, results from the study by Um et al. (2012) revealed that a positive emotional design leads to higher motivation than a neutral design, whereby the authors used the same questionnaire and similar instructional material for both conditions. One explanation could be the methodological difference between these two studies. While our current study, described above, used written text and static pictures, Um et al. (2012) investigated an animated instructional material with spoken text. This difference might have an influence on how the emotional design features affect learner motivation.

### Quality of Answers of the Elaborative Interrogations

It was anticipated that learning with the positive emotional design would lead to qualitatively better answers compared to learning with the neutral or intended-negative emotional design. Contrary to expectations, however, there was no influence of emotional design on the quality of the answers (for similar results, but with another induction procedure see Navratil and Kühl, unpublished). Nevertheless, the quality of the answers to the elaborative interrogations functioned as a positive predictor for the performance of all knowledge test questions. When answering elaborative interrogations, several meaningful processes are performed, i.e., selection of important concepts, thinking about relations, recognition of missing steps, monitoring of the learning process, and re-reading of important text passages. These processes take place during learning and may enhance an overall deeper understanding of the instructional material. Since the specific instruction to answer the elaborative interrogations prompts learners to elaborate on information derived from the instructional material (Roelle et al., 2014; Navratil and Kühl, unpublished), one can deduce that when learners infer this information correctly, they understand the content of the instructional material.

Results of the current study revealed that learner motivation positively predicted the quality of the answered elaborative interrogations as well as the performance on the different knowledge tests, and furthermore that the quality of the answered elaborative interrogations was positively predictive for the performance of all knowledge tests. Results of corresponding mediation analyses revealed that the effect of motivation on the three of the four learning outcome measures – namely for Retention_Interrogation, Retention_NoInterrogation, and Tranfer_Interrogation – could be explained by the performance on the elaborative interrogations. Solely, for Transfer_NoInterrogation the corresponding mediation analysis failed to reach statistical significance. Overall, these results point to the fact that it is not the motivation *per se* that leads to better learning outcomes, but the higher cognitive engagement during learning (as reflected by the quality in answering elaborative interrogations) due to higher motivation.

### Cognitive Load

We hypothesized that cognitive load when learning with elaborative interrogations would differ from when learning by reading. Results revealed no effect with respect to learners’ perceived invested mental effort and concentration. However, the neutral design was perceived as more difficult than the positive emotional design. This result is in line with several other studies (Um et al., 2012; Plass et al., 2014), and one explanation could be that the black and white hues together with a less interesting and more serious neutral instructional material made the material seem more difficult to learn with, compared to the pleasant, richly colored anthropomorphisms-loaded instructional material of the positive emotional design variations. Finally, learning with the positive emotional design led to a higher estimation of likely success in a subsequent knowledge test compared to learning with the neutral design. It might be that learning with a positive emotional design leads to overconfidence in one’s own performance because of positive memories. In another field of work, it has already been shown that positive emotional memories are more susceptible to increased overconfidence than negative emotional memories (Kensinger and Schacter, 2006).

### Learning Outcomes

With respect to learning outcomes, an interaction of emotional design and the SGL activity was anticipated. Learning with the positive emotional design was expected to profit from learning with elaborative interrogations compared to reading only. Regarding both retention scores, no effect either of SGL activity or of emotional design could be revealed. The lack of effect of emotional design is somewhat surprising, as in other studies (Um et al., 2012; Plass et al., 2014) using similar instructional material and learning outcome measurements, an effect of the emotional design was observed; learning with the positive design led to better performances on these questions than when learning with the neutral design. However, it should be pointed out that the instructional materials in these studies were presented as a 7-min program using Flash animation whereas in this study, the whole of the instructional material was presented with text and static pictures. Therefore, the other presentation type may have produced these inconsistencies.

Regarding transfer questions whose concepts had to be generated through elaborative interrogations, results revealed, contrary to expectations that learners learning with a positive emotional design learned better through reading than with the elaborative interrogations. As already noted, the original instructional material was presented in the form of animation and spoken text (Um et al., 2012; Plass et al., 2014), whereas in the current study the instructional material was presented in the form of written text and static pictures. Consequently, one might assume that learning this instructional material might be accompanied by a higher cognitive load than when learning with the original instructional material and its modalities (cf. Kühl et al., 2011). It might also be the case that the positive emotional design implies an increased cognitive load because of the processing of its features which has no negative influence as long as no additional cognitive task is added, like the instruction to answer elaborative interrogations. However, when an additional task is added, it may be the case that the existing cognitive load of positive emotional design can no longer be balanced and a cognitive overload may occur (Plass and Kaplan, 2015), which might lead to reduced learning outcomes. To be able to decide if this is the case, in a further study one could evaluate working memory capacity as a potential moderator. Moreover, it may also be that the discrepancy between the pictures of the positive emotional design and the rather negative learning content may lead to an increased cognitive load.

Unexpectedly, the intended-negative emotional design had generally no effects on the transfer score that addressed concepts asked in the elaborative interrogation questions, although it induced nearly the same emotional state than the positive emotional design. Consequently, it may be expected that a similar effect could be observed when learning with the intended-negative emotional design compared with the positive emotional design. However, when assuming that the unpleasant faces and colors of the intended negative emotional design are less discrepant but rather congruent to the rather unpleasant learning content, this congruency in turn may not lead to an increase in ECL. This may explain why learning with elaborative interrogations was not harmful for the intended-negative emotional design. Hence, for further research the emotional valence of the learning content should be considered, since it can have moderating effects for learning with multimedia design principles (Kühl and Zander, 2017).

With respect to transfer questions which could be derived from the text, a main effect of emotional design could be shown. Learning with an intended-negative emotional design was more beneficial than learning with a neutral design. Furthermore, the mediation analyses showed that this effect was mediated by learner motivation. Since the intended-negative emotional design might lead to higher coherence with the learning topic and higher learning motivation, one might anticipate that this leads to a deeper engagement with the text of the instructional material, which in turn results to a better performance in these transfer questions consequently.

### Limitations/Implications for Further Research

This study is the first attempt to additionally induce a negative emotional state by using emotional design. Although results were not as anticipated, we can replicate the findings of Um et al. (2012) and Plass et al. (2014), that it is possible to induce positive emotional and neutral states by such an internal induction procedure.

Since the intended-negative emotional design differs from the positive emotional design in terms of its mechanisms (such as the influence on transfer knowledge or motivation), but not in its characteristics (such as valence and positive/negative activation; investigated by an emotional state question and the PANAVA-KS), it becomes clear that the impact of emotional designs on emotional states cannot be the only important source that affect learning outcomes (see also Plass et al., 2014). Moreover, since it is not easy to create a negative emotional design, one has to consider which features in a negative emotional design have to be manipulated in order to induce a negative emotional state. For a further study regarding negative emotional design, one could use jagged edges and far more extreme negative baby-face characteristics, such as large noses and small eyes. In order to reduce the visual aesthetics even more for this design, one could also manipulate the essential factor color more extremely, e.g., by selecting unpleasant color combinations against color pairs on the color wheel, reducing contrasts by considering, e.g., lightness or using a high diversity of colors (Moshagen and Thielsch, 2010).

A further point to discuss relates to the questionnaires used as manipulation checks. Possibly, the dimensions of and items used from the PANAVA-KS are not adequate means of distinguishing between the used emotional designs with respect to the instructional material. More precisely, although the positive as well as the intended-negative emotional design leads to rather similar emotional state scores, they showed totally different influences to learner motivation, cognitive load and performance in the knowledge test questions. In addition, one must take into account that the presence of certain adjectives can already lead to a response-shift in emotional state estimation which thus may lead to false results (Howard and Dailey, 1979). One solution could be the use of the Self-Assessment Manikin (SAM; Bradley and Lang, 1994), a non-verbal pictorial instrument to investigate the three-dimensional structure of objects, namely pleasure, arousal and dominance.

In terms of our hypothesis, the inverse interaction of emotional design and learning with elaborative interrogations regarding the transfer learning outcome cannot be ignored. In hindsight one may interpret this finding by theorizing that the characteristics of the positive emotional design in connection with answering elaborative interrogations leads to an increased cognitive load. Because a main effect of answering the elaborative interrogations in combination with an external induction procedure (Navratil and Kühl, unpublished) could already be shown, another induction procedure should be considered to investigate the interaction hypothesis.

## Conclusion

In summary, this study set out to investigate the influence of emotional design on learning with elaborative interrogations. Regarding the use of emotional design features, results revealed that positive emotional design features can induce a positive emotional state. By contrast, the first attempt to construct intended-negative emotional design features was not successful. However, in applying these features, the induced motivation and apparently the coherence between the emotional design features and the learning topic might play a crucial role and should be considered for a further study. It seems also essential to investigate whether other design elements may induce emotional states that influence the learning process. In addition, the quality of the answers to elaborative interrogations functioned as predictor for the performance in the knowledge tests. The supporting processes when learning with elaborative interrogations seem to be meaningful for applying knowledge. Further research should investigate this SGL activity by considering possible moderating variables, to gain a better understanding of how learning with elaborative interrogations works.

Furthermore, it was observable that the combination of positive emotional design features and an additional SGL activity can hamper learning compared to learning by reading. The positive emotional design features may lead to high cognitive load, so that the supporting effect of the elaborative interrogations on deeper understanding cannot function. However, more research is needed on why the combination with elaborative interrogations was not beneficial. The above-mentioned practical considerations could be meaningful impulses for future research.

## Author Contributions

SN substantial contributions to conception, acquisition, collecting, analysis, and interpretation of data for the work and drafting the work. TK substantial contributions to conception and design, analysis, and interpretation of data for the work, revising the work critically and final approval of the version to be published. SH provision of instructional material and helpful remarks for the implementation of this work.

## Conflict of Interest Statement

The authors declare that the research was conducted in the absence of any commercial or financial relationships that could be construed as a potential conflict of interest.

## Footnotes

1 Elaborative interrogations were pre-tested three times till they were clearly formulated based on the learners’ answers and it was ensured that they could be answered.
